# Voltage-Gated Switching of Moiré Patterns in Epitaxial Molecular Crystals

**DOI:** 10.1021/acsnano.4c12708

**Published:** 2024-11-22

**Authors:** Filippo Giovanni Fabozzi, José D. Cojal González, Nikolai Severin, Jürgen P. Rabe, Stefan Hecht

**Affiliations:** a DWI−Leibniz Institute for Interactive Materials, Aachen 52074, Germany; b Department of Chemistry and Center for the Science of Materials Berlin, Humboldt-Universität zu Berlin, Berlin 12489, Germany; c Department of Physics and Center for the Science of Materials Berlin, Humboldt-Universität zu Berlin, Berlin 12489, Germany

**Keywords:** electric field, interfaces, Moiré pattern, scanning tunneling microscopy, epitaxial crystals

## Abstract

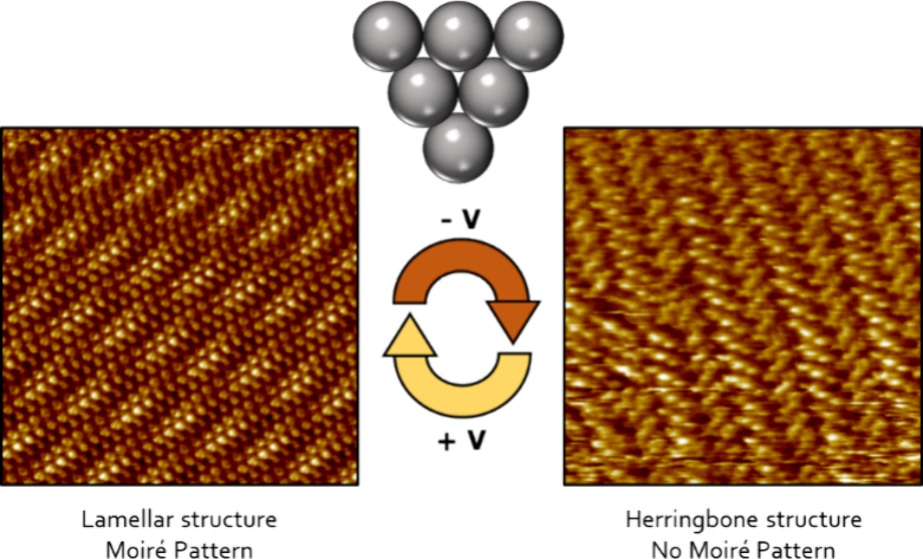

Studying molecular materials at the nanoscale allows us to gain a deeper understanding of supramolecular structure formation and serves as the basis for rationally controlling the resulting interfacial properties. Here, we describe the formation of extended Moiré patterns resulting from the assembly of dipolar π-conjugated molecules on highly oriented pyrolytic graphite at the liquid–solid interface as characterized by scanning tunneling microscopy (STM). By switching the bias of the sample and thus the orientation of the external electric field in the vicinity of the STM junction, structural reorganization of the molecular building blocks and the resulting organic 2D crystal is induced and can conveniently be monitored *in situ* by the appearance and disappearance of the Moiré patterns. Importantly, the formation and loss of the Moiré patterns are fully reversible, providing exquisite control over epitaxial molecular crystals. Our approach provides fundamental insights into the supramolecular organization and resulting superstructure formation of incommensurable 2D lattices upon applying an electric field and enables the rational tuning of Moiré patterns as a key step toward the potential integration of organic 2D crystals in molecular nanodevices.

## Introduction

Molecular epitaxial crystals are a class of ultrathin materials composed of molecular arrays with uniform in-plane orientation and long-range order.^1−3^ The assembly process carried out generally on crystalline surfaces^4,5^ is mainly driven by molecule–molecule^6^ and molecule–substrate interactions.^7,8^ These interactions include van der Waals (vdW) interactions, hydrogen^9−12^ and halogen^13−16^ bonding, as well as π–π stacking.^17−19^ Meanwhile the concentration, diffusion, and nature of the substrate^20^ are crucial parameters to be considered to develop defect-free extended nanostructures for scale-up applications.^21,22^

A peculiar phenomenon of two or more superimposed 2D crystals is the emergence of specific interferences originating from a mismatch between crystal lattices at the interface, leading to the formation of distinct new topologies more generally called Moiré patterns.^23,24^ At the atomic level, they are a result of the incommensurability between 2D lattices.^25,26^ Moiré patterns in layered inorganic vdW 2D materials,^27^ such as twisted bilayer graphene,^28−30^ are formed when a mismatch in the orientation is given, for example, induced by mechanically twisting the 2D layers. Tuning of the twisting angle modifies the electronic coupling, leading to the formation of new intriguing electronic structures. This is manifested in the appearance of flat bands and unconventional superconductivity^31,32^ or leading to diametrically opposite insulating properties.^33−35^

In molecular epitaxial crystals, the observation of Moiré patterns is a powerful tool to probe the stability of supramolecular networks as well as define lattice parameters with unprecedented precision.^36^ However, Moiré patterns for organic–inorganic vdW heterostructures gained only little attention due to the difficulty to experimentally investigate their physical and chemical properties. Surprisingly, tuning Moiré patterns at the nanoscale in organic epitaxial crystals has remained unexplored, despite the fundamental interest in engineering and modulating the electronic properties in molecular 2D crystalline materials.

Considering that on-surface supramolecular structures are generally controlled either by adding a new component to the system^37,38^ or by using heat (annealing),^39,40^ light,^41^ or electric fields,^42,43^ the latter presents distinct advantages as it is reversible and tunable in strength (potential) and direction (bias) as well as easy to operate. For example, networks formed by H-bonding of carboxylic acids can easily be influenced by switching the direction of the applied electric fields.^44−46^ Important advances regarding the understanding of the switching mechanism have been made using density functional theory calculations.^47^

Scanning tunneling microscopy (STM) under ambient conditions at the liquid–solid interface^26,48^ is an established scanning probe microscopy (SPM) technique employed for nanomaterial characterization with submolecular precision. STM can be considered analogous to a capacitor because the tunneling current is measured between a sharp metallic tip and a conductive surface in close proximity after application of an electric field, resulting in imaging down to submolecular resolution.^49^ Recently, this technique has also been used as an activation tool to locally induce molecular reactivity^50−52^ and to control supramolecular structures in self-assembled molecular networks (SAMNs)^39,45,53^ with nanoscale precision.

**Figure 1:**
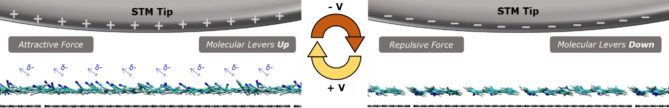
Switching 2D molecular crystals by manipulation of the electric fields in the STM junction: A positive tip bias attracts the negatively polarized CN-groups (left), whereas a negative tip bias leads to repulsion and supramolecular reorganization (right).

Here, we report and investigate the supramolecular switching process of a stilbene derivative, which has been largely exploited as an optical material in thin film applications.^54−56^ Notably, the investigated molecular system forms a Moiré pattern on highly oriented pyrolytic graphite (HOPG) at both the liquid–solid and air–solid interface caused by the incommensurability of the self-assembled molecular layer with the substrate surface. The presence of out-of-plane polarized CN-groups, functioning as submolecular “levers”, allows for the modulation of the supramolecular organization (SAMN structure) by means of tuning the external electric fields (EEFs) in the STM junction (Figure 1). The thus demonstrated controlled and robust switching of Moiré patterns in organic 2D crystals represents an appealing approach to realize tunable next-generation molecular devices.

## Results and Discussion

### Synthesis and On-Surface Formation of Molecular Epitaxial Crystals

**Figure 2:**
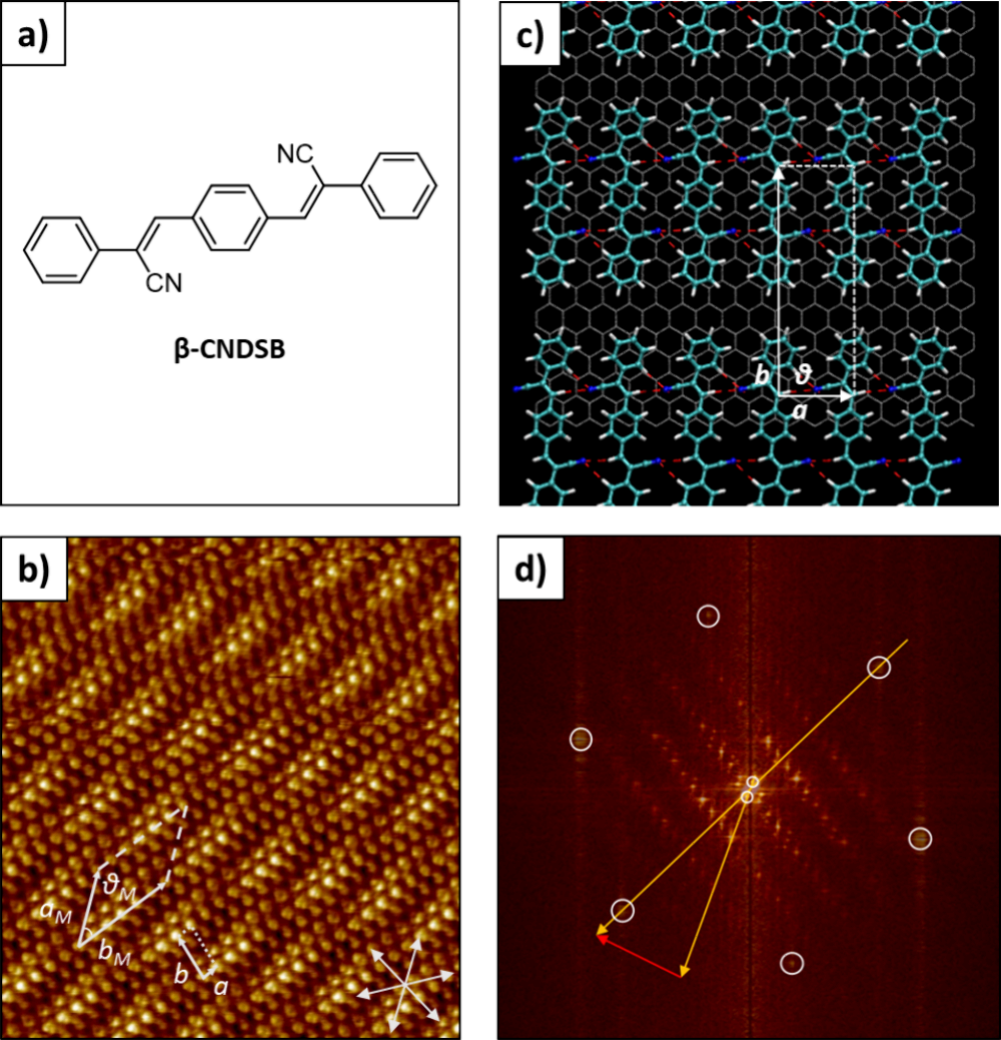
(a) Chemical structure of **β-CNDSB**. (b) HR-STM image acquired at the liquid–solid interface (octanoic acid–HOPG) of a molecular network formed by **β-CNDSB**. The six arrows at the bottom right represent the HOPG substrate axes. Scanned area: 20 × 20 nm, *I* = 150 pA, and *V* = 900 mV. (c) Molecular model as a result of energy minimization of the **β-CNDSB** molecular network. (d) Superposition of respective 2D-FFTs of panel b and the HOPG imaged subsequently. The orange arrows connect the frequencies of the HOPG (larger) and molecular network (smaller). The red arrow results from the difference of the two and represents the angle between **β-CNDSB** molecules and graphite axis.

As a starting point, the formation and switching properties of an organic 2D crystal composed of a *para*-phenylenevinylene dimer, i.e., 1,4-bis(2′-cyano-2′-phenylethenyl)benzene (**β-CNDSB**), was investigated (Figure 2a). Given the extended π-conjugated aromatic backbone that results in efficient luminescence,^54^ studies of the molecular organization in (ultra)thin films are relevant for the potential integration into hybrid organic–inorganic (opto)electronic devices.

**β-CNDSB** was obtained via a twofold Knoevenagel condensation between commercially available terephthaldehyde and benzyl cyanide using CsOH as the basic catalyst (see the Experimental Section for further details).^57^ Preparation of the organic 2D crystal was carried out by drop casting a solution of **β-CNDSB** (10^–5^ M in EtOH) on freshly cleaved highly HOPG. STM measurements were carried out under ambient conditions at the liquid–solid interface. STM imaging shows the successful formation of a **β-CNDSB** SAMN at the octanoic acid-HOPG interface (Figure 2b). Atomic force microscopy (AFM) shows a rather homogeneous extended soft layer that presents a step height of around 0.8 nm, as confirmation of the mono-to-bilayer formation (Figure S1).

### Characterization of the Moiré Pattern in Molecular 2D-Crystals

High-resolution STM images acquired at the liquid–solid interface (octanoic acid–HOPG) reveal that **β-CNDSB** molecules are organized in a lamellar structure (Figure 2b). The experimentally determined unit cell parameters of *a* = 0.70 ± 0.1 nm, *b* = 2.20 ± 0.2 nm, and θ = 93 ± 2° are in good agreement with the corresponding molecular model obtained by energy minimization of a simulated molecular network on a constructed graphitic surface (Figure 2c and Figure S13) giving a calculated unit cell of *a* = 0.70 nm, *b* = 2.13 nm, and θ = 89°. Analysis of the molecular model reveals H-bonding contacts between the vinylic hydrogen atoms and nitrogen atoms of the CN-groups. To provide a better understanding on the displacement of single molecules to the reader, simulated **β-CNDSB** was placed on top of an HR-STM image (Figure S2). For further details, HOPG used for Figure 2a calibration and the respective 2D-FFTs are reported in Figure S3.

Visual inspection of the STM image exemplified in Figure 2b shows periodic variation of contrast attributed to the formation of a Moiré pattern, revealing Moiré lattice parameters of *a*_M_ = 5.38 ± 0.1 nm, *b*_M_ = 4.41 ± 0.2 nm, and θ_M_ = 29 ± 2°, composed of seven molecules per single unit cell. The difference in contrast characterized by “bright” and “dark” spots originates from the incommensurability between the molecular layer and the substrate and is attributed to an inhomogeneous electronic coupling of individual **β-CNDSB** molecules to the HOPG, resulting in a variation of the apparent height of the molecules (Figure S4).

In the context of layered Moiré molecular 2D crystals, the periodicity determines the electronic and optical properties of 2D stacked materials. Here we derive a model based on the experimental results that describes the Moiré periodicity (*d*_Moiré_) in our system (eq 1):
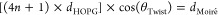
1

To validate eq 1, we estimated the distance between two molecules to correspond to four zigzag rows of graphite (see the molecular model in Figure S14). Considering an entire Moiré unit cell of *n* = 7 and the experimental twisting angle between **β-CNDSB** molecules and the HOPG axis of θ_Twist_ = 29° (Figure 2d), the Moiré periodicity can be calculated as *d*_Moiré_ = 5.40 nm, which is in excellent agreement with the obtained experimental STM images. A more detailed graphical explanation of eq 1 is provided in Figure S5.

As the measurements were carried out at the liquid–solid interface, it is important to know whether the obtained Moiré pattern originates solely from the **β-CNDSB** 2D molecular network or involves coadsorbed octanoic acid molecules. To rule out the latter scenario, we performed imaging of dry samples. The STM images in the absence of octanoic acid revealed the identical unit cell and Moiré pattern as when the solvent was present (Figure S6). Therefore, we assume that a direct influence of the octanoic acid on the Moiré pattern formation can be omitted. Moreover, no substantial differences were found regarding defects and domain distribution.

Moreover, it is important to verify whether the obtained Moiré pattern is a thermodynamically stable state or a metastable intermediate state. Therefore, the sample was heated *ex situ* up to 80 °C for ∼1 h and then characterized by STM at both the liquid–solid (octanoic acid–HOPG) and air–solid interfaces. The obtained STM images (Figure S7) show no structural differences when compared to the original results described above. Based on these findings, we attribute the obtained Moiré pattern to represent the thermodynamic minimum structure.

### Switching of the Moiré Pattern Induced by the STM Tip

**Figure 3:**
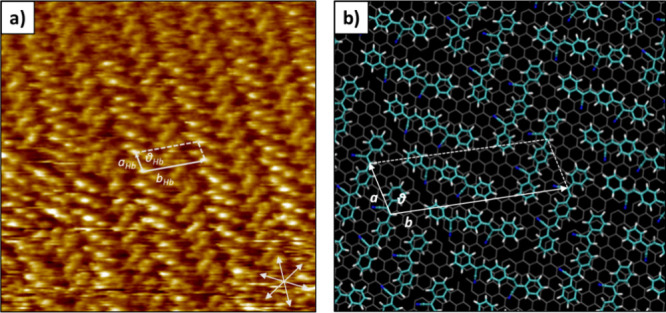
(a) HR-STM image measured at the liquid–solid interface (octanoic acid–HOPG) obtained after switching from positive to negative tip bias showing the herringbone supramolecular structure. The six arrows at the bottom right represent the HOPG symmetry used for image calibration. Scanned area: 16 × 16 nm, *I* = −131 pA, and *V* = −900 mV. (b) Molecular model of the herringbone 2D molecular network.

Surprisingly, upon changing the tip bias used for STM imaging, we observed a different supramolecular organization (Figure S8). It is important to mention that no STM tip pulses were needed to visualize the newly formed 2D molecular network. High-resolution STM images obtained at the octanoic acid–HOPG interface reveal a herringbone-like structure of **β-CNDSB** 2D molecular networks with experimental constant lattice parameters of *a*_Hb_ = 1.04 ± 0.1 nm, *b*_Hb_ = 3.02 ± 0.2 nm, and θ_Hb_ = 83 ± 2° characterized by two molecules per single unit cell (Figure 3a). The experimental values are in good agreement with the molecular model in Figure 3b that shows calculated unit cell parameters of *a*_Hb_ = 0.98 nm, *b*_Hb_ = 2.98 nm, and θ_Hb_ = 90°. Herein, **β-CNDSB** forms a less dense network reflected in a surface coverage of 0.686 nm^–2^ compared to the 0.736 nm^–2^ of the lamellar conformation and characterized by a high presence of multiple domain boundaries and defects, presumably due to a rather low molecular mobility (Figures S9 and S10). The obtained supramolecular structure appears repeatedly in different scanned areas, obviously attributed to the stabilization of the herringbone organization induced by the negative tip bias. Moreover, the formation of the herringbone 2D network was observed only by switching the tip bias at the liquid–solid interface, clearly pointing to a desorption–adsorption mechanism. Additionally, STM analysis revealed identical behavior by the **β-CNDSB** on HOPG if the measurements were started directly at the negative tip bias, revealing an immediate formation of the herringbone supramolecular structures.

**Figure 4:**
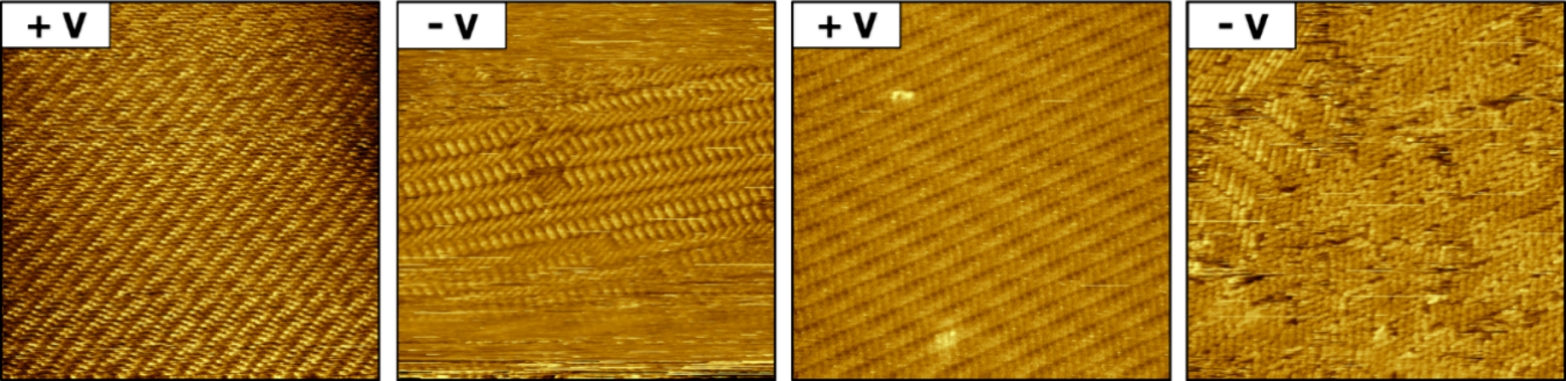
Two switching cycles measured by STM at the liquid–solid interface (octanoic acid–HOPG) starting with positive tip bias (+V), where the lamellar structure was observed, while at negative tip bias (−V) the herringbone structure was formed. After switching back to +V, the Moiré pattern was always recovered. Scanned area: 30 × 30 nm; for positive bias (+V): *I* = 131 pA and *V* = 900 mV; and for negative bias (−V): *I* = −131 pA and *V* = −900 mV.

The reversible interconversion of the two supramolecular structures was demonstrated by switching back and forth the STM tip bias, i.e., from positive to negative and from negative to positive (Figure 4), resulting in a controllable transition between the lamellar structure (with Moiré pattern) and the herringbone structure (without Moiré pattern). Starting from a positive tip bias, multiple voltage cycles were performed by alternating between +900 and −900 mV while the reorganization was continuously monitored by STM at the octanoic acid–HOPG interface. The lamellar structure was always fully recovered without the appearance of any defects within the supramolecular network. In contrast, the herringbone structure showed differing reorganized structures characterized by a high defect density and multiple domain boundaries. Supramolecular reorganization was followed by continuously scanning at constant negative bias (Figure S10), and an Ostwald ripening process was observed, giving rise to a slow defect healing process. Unfortunately, imaging at negative tip bias proved extremely challenging due to instrument instabilities. Therefore, switching from positive to negative tip bias required a longer time, often accompanied by drift during the first scanning. Instead, switching from negative to positive tip bias resulted in a rather easy imaging process of the 2D networks.

To obtain a better understanding regarding the effect of the high electric fields induced by the STM tip on the 2D molecular network, incremental changes in the tip bias were applied as well. Starting from +1.4 V, the STM tip bias was slowly decreased until the underlying HOPG was visible (Figure S11). No substantial changes in the contrast were revealed during imaging. Note that no other supramolecular organization was visualized by varying the bias, excluding a multilayered nature of the 2D molecular network under these conditions.^58^ At negative tip bias, the optimal imaging conditions at **–**0.9 V revealed the formation of the herringbone structure, as expected. Incremental changes up to **–**0.6 V resulted in decreasing image contrast yet with the 2D networks still visible. At **–**0.4 V, no supramolecular structures could be visualized anymore. STM tip cleaning pulses (at either **–**2 or **–**4 V) could not increase the resolution (Figure S12).

### Mechanism of the Supramolecular Switching Induced by the STM Tip

Based on the experimental observations, we hypothesize that the orientation of the negatively charged CN-groups, serving as submolecular levers, can be controlled by manipulation of the electric field induced by the STM tip. While a positive tip bias results in an attractive force that twists the levers parallel to the electric field, a negative tip bias results in a repulsive force that forces the levers to be perpendicular to the electric field (Figure 1).

To gain a more detailed understanding and verify/falsify our above hypothesis regarding the stability of the two supramolecular structures upon the application of an electric field, molecular dynamics (MD) simulations were performed. As initial configuration, 24 molecules of **β-CNDSB** were placed in a *transoid* conformation with a starting unit cell as shown in Figure 2c and at a distance of ∼3.4 Å to a graphene sheet (Figure S14a). After 1 ns at 300 K, the resulting structure is composed of a more densely packed unit cell with the cell parameters of *a* = 0.69 nm, *b* = 1.91 nm, and θ = 83° (Figure S14b), in good agreement with the STM results and the proposed molecular model (Figure S13). Molecular dynamics calculations show that individual **β-CNDSB** molecules adopt a slightly twisted structure, with the polarized CN-groups pointing out of the graphite basal plane.

**Figure 5:**
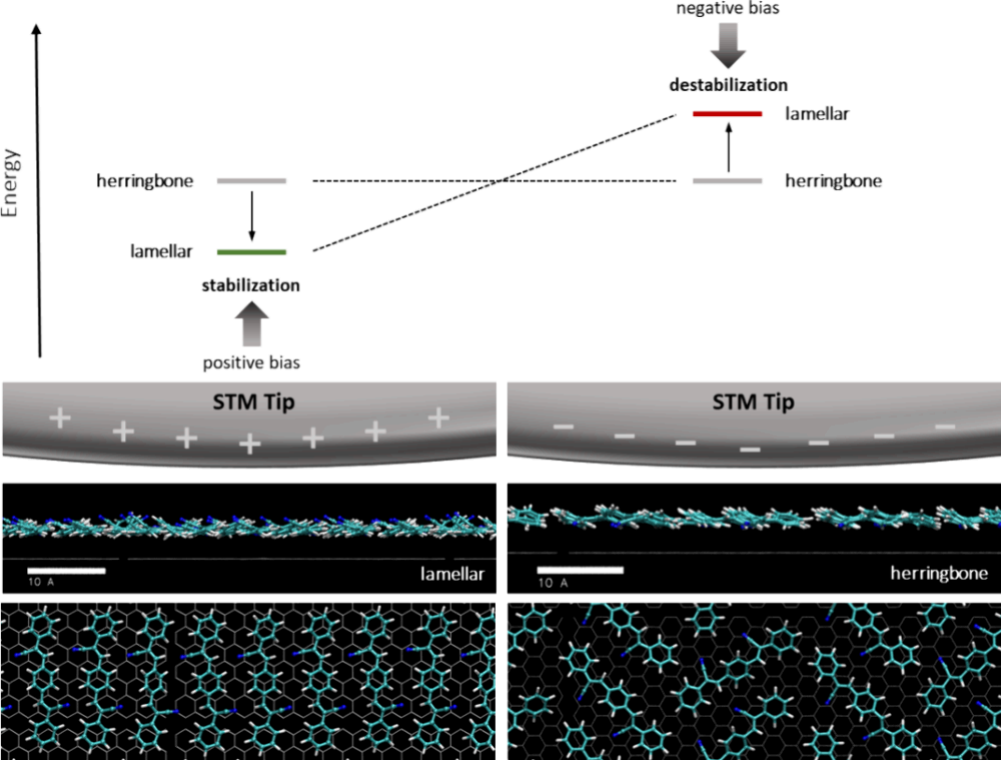
Qualitative representation of the stabilization and destabilization processes induced by tuning the electric field induced by the STM tip. A positive tip bias results in a stabilized lamellar conformation, while a negative tip bias results in a destabilized lamellar conformation, thus leading to a preference for the alternative herringbone structure (top). Snapshots of the molecular dynamics simulation when a negative electric field (left) and a positive electric field (right) are applied, showing the polarized CN-groups pointing away and toward the substrate, respectively.

For the lamellar structure (Figure 2), a negative electric field — analogous to a negatively charged substrate and a positively charged tip — is applied perpendicular to the substrate. Application of an electric field of −1.5 V/nm during MD simulations causes a pronounced tilting of the negatively polarized CN-groups, i.e., the molecular levers, pointing toward the positively charged tip (Figure 5 left and Figures S15 and S17).

A stable herringbone network could only be obtained in the simulations after the application of a positive electric field and with individual **β-CNDSB** molecules adapting a *cisoid* conformation with both CN-groups pointing in the same direction (Figure S17). On the contrary, a herringbone network mostly composed of *transoid***β-CNDSB** molecules rearranges into a lamellar structure (Figure S16). Furthermore, under the influence of a negatively charged tip, the CN-groups reorient toward the substrate as a result of the repulsive force induced by the positive electric field (Figure 5 right). As for the experimental results shown in Figure 4, also here, switching of the electric field from positive to negative led to a recovery of the lamellar structure (Figure S18).

Dissociation energies for individual **β-CNDSB** in both supramolecular structures were determined theoretically by simulations. By incrementally shifting a **β-CNDSB** molecule from the constructed graphene plane, the dissociation energy was calculated as the difference in potential energy between each step and the initial configuration. As a result, for the lamellar conformation, a dissociation energy of *E*_lam_= 204 ± 7 kJ/mol was calculated, whereas for the herringbone structure, it amounts to *E*_Hb_= 208 ± 3 kJ/mol. Moreover, when a negative electric field of −1.5 V/nm is applied, the dissociation energy of the **β-CNDSB** in the lamellar structure amounts to *E*_lam–_= 209 ± 7 kJ/mol, while when a positive electric field of +2 V/nm is applied, the dissociation energy for the herringbone structure amounts to *E*_Hb+_= 205 ± 5 kJ/mol (Figure S19).

The combination of experimental and computational results suggests that it is possible to selectively stabilize or destabilize a specific supramolecular structure, i.e., the lamellar arrangement, able to interact with the electric field via out-of-plane oriented polarized CN-groups (Figure 5). By applying strong electric fields, it is therefore possible to switch between two different supramolecular arrangements and even their associated superstructures, i.e., Moiré patterns.

## Conclusions

The formation of organic epitaxial crystals of a π-conjugated molecular system (**β-CNDSB**) was investigated by room temperature STM at the liquid–solid interface. Deposition of **β-CNDSB** on HOPG led to the emergence of long-range Moiré patterns originating from the incommensurability between the supramolecular network and the graphite lattices. Tuning of the STM tip bias led to supramolecular reorganization by destabilizing the lamellar structure (with a Moiré pattern) and giving rise to a herringbone structure (without Moiré pattern). The process is reversible and thus allows for convenient control over Moiré pattern formation in 2D supramolecular structures. STM imaging combined with molecular modeling and dynamics simulations provided detailed insight into the switching mechanism induced by the alternating applied electric field. It appears that the latter interacts strongly with the polarized CN-groups of the molecule and leads to supramolecular structure formation that favors their proper orientation.

In addition to a thorough understanding of the underlaying structural features, our rather practical approach to switch 2D supramolecular structures containing Moiré patterns using the electric field in a capacitor setup would enable the tuning of electronic properties in noncovalent materials with the possibility to integrate such systems in hybrid nanodevices. Along these lines, future efforts will be dedicated to broadening the scope of our concept by applying it to other 2D materials such as transition metal dichalcogenides and to understanding how changes induced in the supramolecular structure of epitaxial molecular crystals lead to a change in the electronic properties of layered materials.

## Experimental Section

### Synthesis of 1,4-Bis(2′-cyano-2′-phenylethenyl)benzene (**β-CNDSB**)

Terephthaldehyde (100 mg, 0.746 mmol) and benzyl cyanide (177 mg, 1.51 mmol) were dissolved in 5 mL of EtOH. CsOH (10 μL, 50 wt % in water) was added, and the reaction mixture was heated to reflux for 2 h until a yellow precipitate was formed. After cooling to room temperature, the precipitate was filtered off, washed with EtOH, and dried *in vacuo*, affording **β-CNDSB** as a bright yellow powder (0.245 mg, 98%). ^1^H NMR: (400 MHz, DMSO-*d*_*6*_) δ = 8.13 (s, 2H), 8.09 (s, 4H), 7.81 (d, J = 7.2, 1.8 Hz, 4H), 7.61–7.44 (m, 6H) ppm.

ESI + MS *m*/*z* calculated for C_24_H_16_N_2_: 333.14, found: 333.1394.

### Sample Preparation

A droplet of a **β-CNDSB** solution in EtOH (10^–5^ M, 5% DMSO) was casted onto a freshly cleaved highly oriented pyrolytic graphite (HOPG) substrate, and the solvent naturally evaporated after a few minutes at room temperature. Thermal annealing of the sample for 5 min at 70 °C was used to completely remove the EtOH from the surface. Afterward, a droplet of octanoic acid was applied for STM measurements at the liquid–solid interface.

## Supporting Information Available

Materials and methods; scanning probe microscopy measurements; molecular modeling and dynamics; and NMR characterization (PDF)
