# Supplementary material for: Voltage-Gated Switching of Moiré Patterns in Epitaxial Molecular Crystals
Source: ACS Nano. 2024 Nov 22;18(49):33664–70. doi: 10.1021/acsnano.4c12708 (PMC11636263; doi:10.1021/acsnano.4c12708)
Supplement: Supplementary file 1 — nn4c12708_si_001.pdf [file nn4c12708_si_001.pdf]

Supporting Information

Voltage-Gated Switching of Moiré Patterns in Epitaxial Molecular Crystals

Filippo Giovanni Fabozzi,[a,b] José D. Cojal González,[c] Nikolai Severin,[c] Jürgen P. Rabe,[c] and Stefan Hecht[a,b]*

[a] DWI – Leibniz Institute for Interactive Materials, 52074, Aachen, Germany

[b] Department of Chemistry and Center for the Science of Materials Berlin, Humboldt-Universität zu Berlin, 12489, Berlin, Germany

[c] Department of Physics and Center for the Science of Materials Berlin, Humboldt-Universität zu Berlin, 12489, Berlin, Germany

Correspondence to: sh@chemie.hu-berlin.de

Table of Contents

1. MATERIALS AND METHODS ---------- 2

2. SCANNING PROBE MICROSCOPY MEASUREMENTS ---------- 4

3. MOLECULAR MODELLING AND MOLECULAR DYNAMICS ---------- 12

4. NMR CHARACTERIZATION ---------- 17

5. REFERENCES ---------- 18

1. Materials and Methods

Starting materials and reagents were used as received from Sigma-Aldrich, TCI, ABCR or Fisher Scientific without further purification. NMR spectra were recorded on a Bruker DPX 300 Spectrometer (300 MHz for ^{1}H). ^{13}C for **β-CNDSB** could not be measured due to the very low solubility of the compound in the common deuterated solvents. For UPLC-MS a Waters UPLC Acquity with a Waters Alliance System (Waters Separations Module 2695, Waters Diode Array Detector 996 and Waters Mass Detector ZQ 2000) was used. HR-MS measurements were performed on a Xevo G3 QTof. Weighing of small quantities was performed on a Sartorius ME5 analytical microbalance.

SPM Measurements

Scanning Tunneling Microscopy (STM) measurements were carried out using a NaioSTM system (Nanosurf, Switzerland) operating in constant-current mode at room temperature under ambient conditions. STM tips were prepared by mechanical cutting of a Pt/Ir (80/20) wire (Goodfellow Cambridge Ltd.). Fresh graphite surfaces were prepared by mechanical cleaving highly orientated pyrolytic graphite (HOPG, Bruker, 12 mm x 12 mm x 2 mm, ZYB Grade). STM measurements were carried out either at the solid-liquid (HOPG-octanoic acid) or solid-air interface. Atomic Force Microscopy (AFM) characterizations were performed using an AFM Multimode 8 (Bruker Corporation), software Nanoscope 9.4 with SCANASYST-AIR-HR probes: f_0= 130 kHz; k= 0.4 N/m; T= 0.5 µm. AFM characterizations were carried out by using ScanAsyst-Mode (Bruker Corporation). AFM images were flattened by using a second order plane correction and the height was measured by using the tool for cross section in Gwyddion[1] (Czech Metrology Institute, Brno, CZ). Data and Image analysis were carried out by SPIP (Image Metrology A/S, Lyngby, DK). STM images shown in the main text were corrected for piezo drifts, besides particular cases (switching cycles in Figure 4). To do this, an image of graphite was taken immediately after acquisition of an image with molecules. The correction factors were calculated assuming graphite unit cell to be hexagonal with a lattice parameter of 0.246 nm. High resolution STM images shown in the manuscript were cropped from a bigger calibrated image.

Molecular Modelling and Molecular Dynamics

Molecular Modelling calculations were carried out with Material Studio using the *pcff* force-field with a cut-off distance of 0.95 nm and with periodic boundary conditions imposed. Single molecules were placed manually close to a surface of graphite constructed of two graphene planes. H-bonds were calculated with a maximum distance of 2.3 Å and represented with light blue dotted lines. Energy minimization was run with an ultra-fine convergence level (20000 interactions). Molecular dynamics simulations were carried out using GROMACS 2020.2[1] package.[2] All bonds, angles, dihedrals and non-bonded terms are parametrized using CHARMM general force field C36.[3,4] The structures were first minimized using a steep descendent integrator with a force convergence criterium smaller than 100 kJ mol^{-1} nm^{-1}. NVT runs were carried out for production of the data with a time step of 1 fs. Canonical sampling was assured by using a velocity rescale thermostat[5] at 250/300 K. Bonds were hydrogen is involved were constrained using the LINCS

solver. The cut-off distance for non-bonded interactions was set to 1.5 nm. To minimize edge effects, we applied periodic boundary conditions with a minimum image convention, meaning that only the nearest image is considered for short-range non-bonded interactions.

To compute the dissociation energy, a representative frame was first extracted from MD trajectories, ensuring it corresponded to a local minimum for different system conditions and configurations. Five molecules were then selected, and each was incrementally shifted by 0.2 Å per step over 80 steps, with energy minimization performed at each step to eliminate local strain. The dissociation energy was calculated as the difference in potential energy between each step and the initial step (0 Å displacement), allowing the construction of dissociation energy plots. The average dissociation energy was taken over the last 30 steps for the five molecules, and the error was estimated as the standard deviation of potential energies over these steps. The calculations were performed using GROMACS 2020.2, employing the steepest descent algorithm with a force tolerance of 100.0 kJ/mol/nm and a step size of 0.01 nm, up to a maximum of 500,000 steps.

2. Scanning Probe Microscopy Measurements

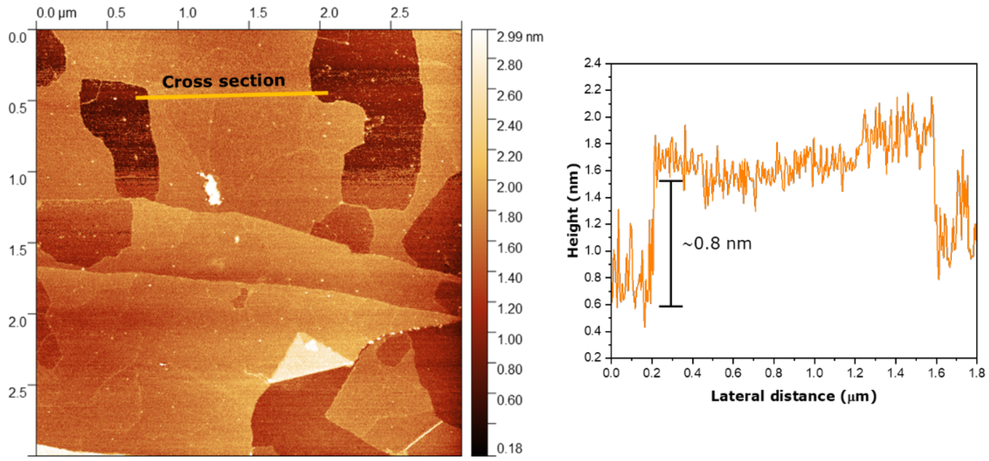

Figure S1. Atomic Force Microscopy image showing the overall domain distribution and surface coverage of **β-CNDSB**. The orange cross section reported in the graph on the right reveals a step height of around 0.8 nm as confirmation of mono-to-bilayer formation.

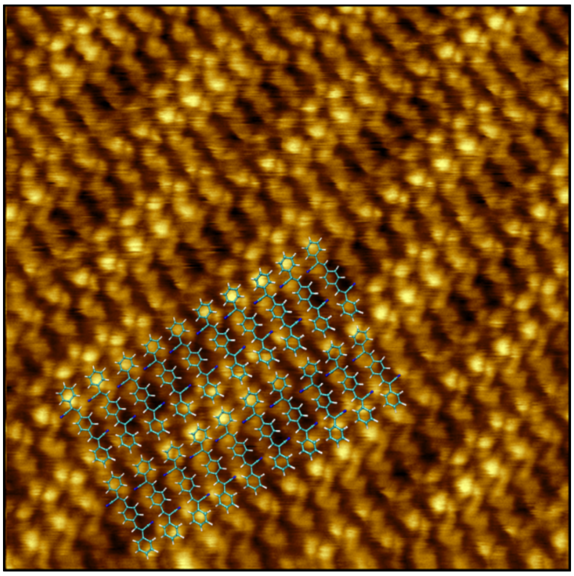

Figure S2. HR-STM image of the lamellar supramolecular structure formed by **β-CNDSB**. Single molecules obtained by molecular modelling are placed on top of the STM image in order to precisely show the organization and correctly assign the structure. Scanned area: 10 nm x 10 nm; *I* = 171 pA, V = 900 mV.

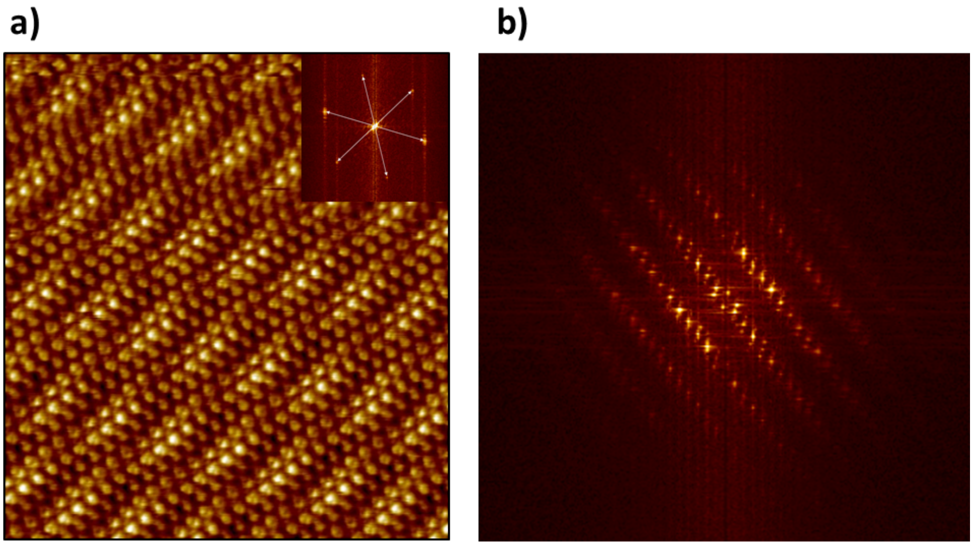

Figure S3. **a)** HR-STM image of the SAMN reported in **Figure 2b**, in the main text. In the inset is reported the HOPG used for calibration. **b)** 2D-FFT of the molecular network. Scanned area: 20 nm x 20 nm; *I* = 150 pA, V = 900 mV

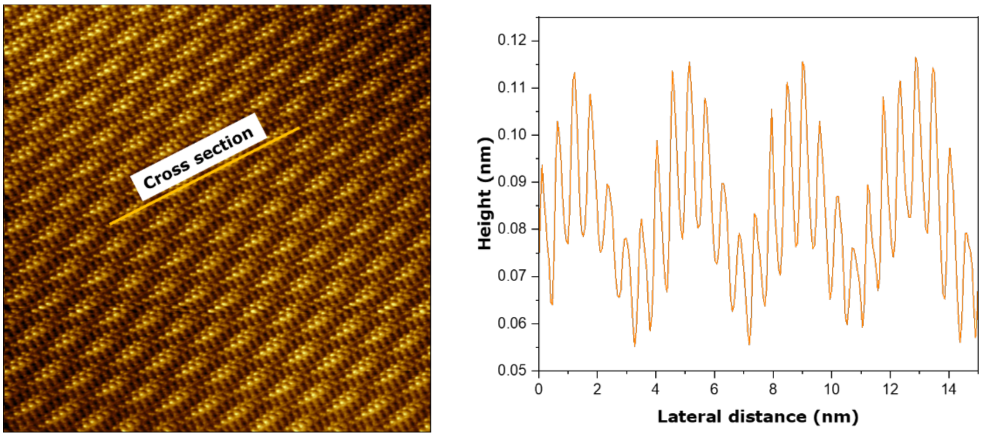

Figure S4. STM image of the 2D molecular network of **β-CNDSB**, measured at the liquid-solid interface (octanoic acid-HOPG). As showed in the graph on the right, the Moiré Pattern is characterized by a difference of step height originated from the incommensurability between single molecules of **β-CNDSB** and the graphitic surface. Scanned area: 30 nm x 30 nm; *I* = 150 pA, V = 900 mV

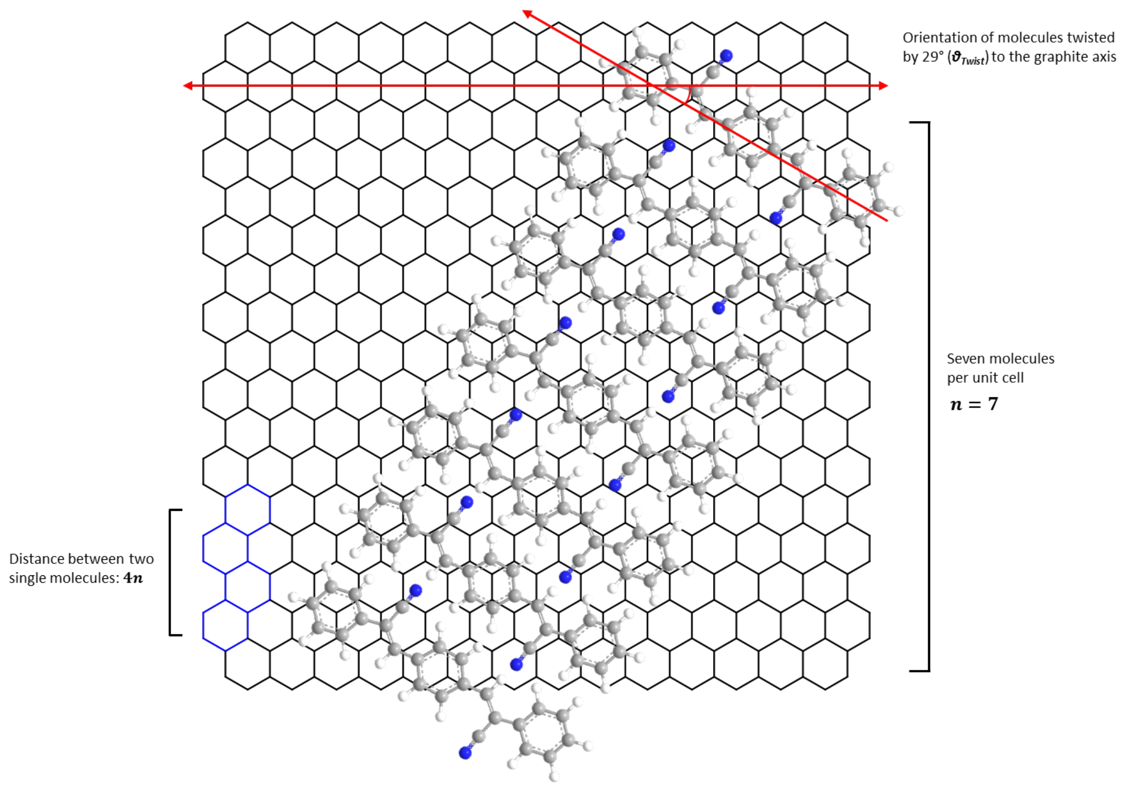

Figure S5. Schematic representation of **Equation 1** reported in the main text. The hexagonal lattice (in black) represents the graphene layer directly below the adsorbate (**β-CNDSB**). Seven molecules that correspond to a single Moiré repeating unit are disposed in the lamellar conformation. The blue highlighted hexagons represent the distance between two single molecules of **β-CNDSB** i.e. four graphitic rows per two molecules. The red lines on top represents the orientation of the graphite and the molecules. From the superpositions of the FFTs resulted from the STM images of **β-CNDSB** molecular network and HOPG, the rotation of the two orientations is quantify as 29° ($\boldsymbol{\theta_{Twist}}$). By substituting the values, **Equation 1** resulted in 5.40 nm.

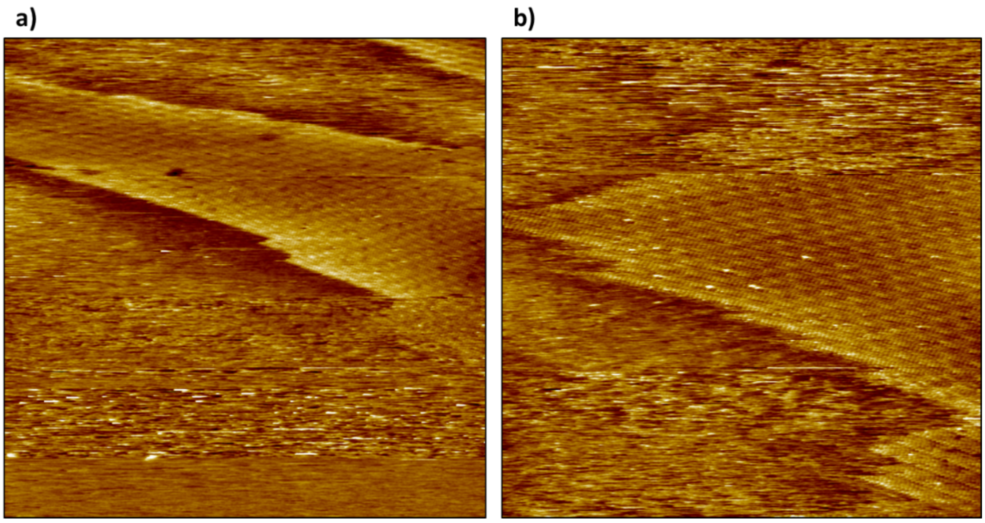

Figure S6. STM images acquired at the air-solid interface on HOPG. The molecular networks present the same organization with visible Moiré Pattern as for the STM measurements carried out at the liquid-solid interface (octanoic acid-HOPG). Scanned areas: a) 80 nm x 80 nm, b) 50 nm x 50 nm. *I* = 131 pA, V = 900 mV

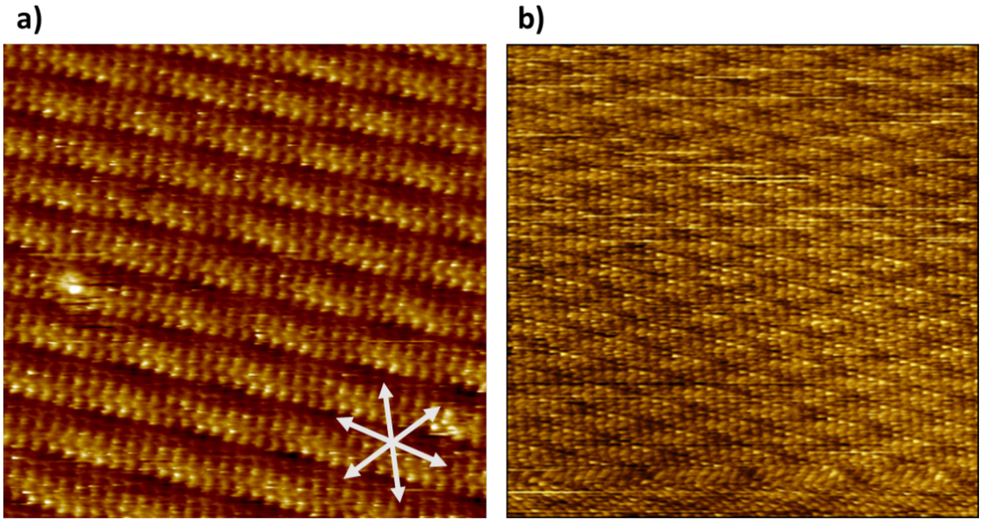

Figure S7. Two representative STM images of SAMN obtained by drop casting of a solution of **β-CNDSB** (10^{-5} M in EtOH, 5% DMSO) after thermal annealing at 80 °C for (up to) 1 hour. **a)** Measured at the liquid-solid interface (octanoic acid-HOPG). Scanned area: 25 nm x 25 nm; *I* = 150 pA, V = 900 mV **b)** STM image acquired at the solid-air interface. Scanned area: 25 nm x 25 nm. *I* = 119 pA, V = 900 mV. The Moiré pattern is still clearly visible in both conditions.

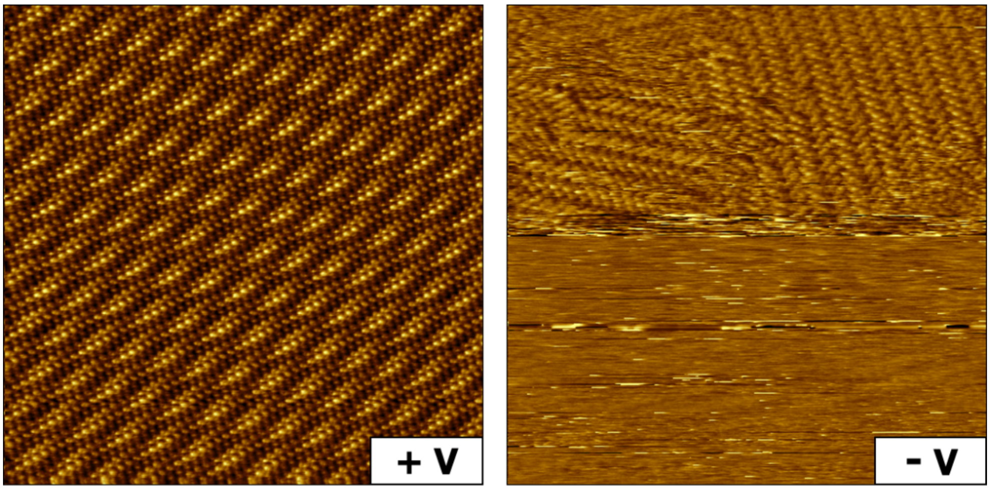

Figure S8. Example of switching process of the 2D molecular network of **β-CNDSB**. The tip bias was tuned from positive (on the left, measured at +900 mV) to negative (on the right, measured at -900 mV) and monitored by STM at the liquid-solid interface (octanoic acid-HOPG). The new supramolecular assembly (herringbone) of **β-CNDSB** was visible only after few scans after switching the tip bias from positive to negative bias, due to the molecular re-organization. Scanned areas: 30 nm x 30 nm; Positive tip bias: I = 131 pA, V = 900 mV, Negative tip bias: I = — 131 pA, V = — 900 mV

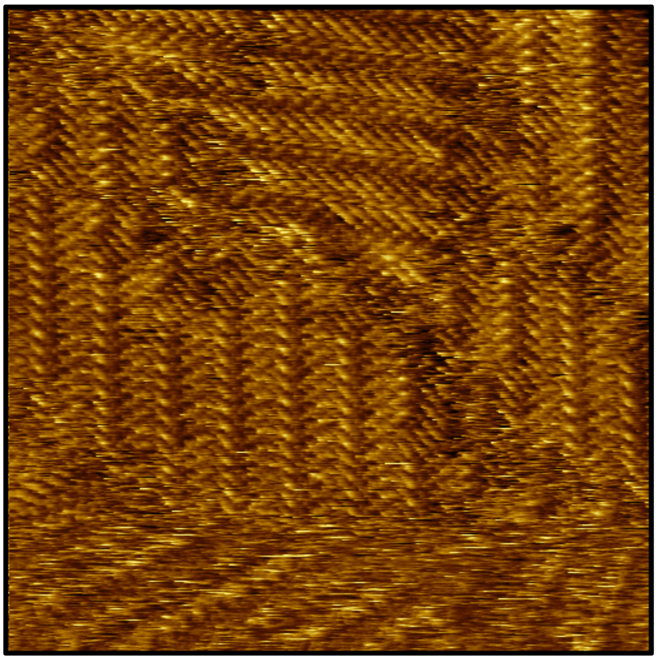

Figure S9. Supplementary STM image acquired at the liquid-solid interface (octanoic acid-HOPG) by using negative tip bias. The developed herringbone structure presents high degree of defects and short-range order. Scanned area: 30 nm x 30 nm; I= -131 pA, V= -900 mV.

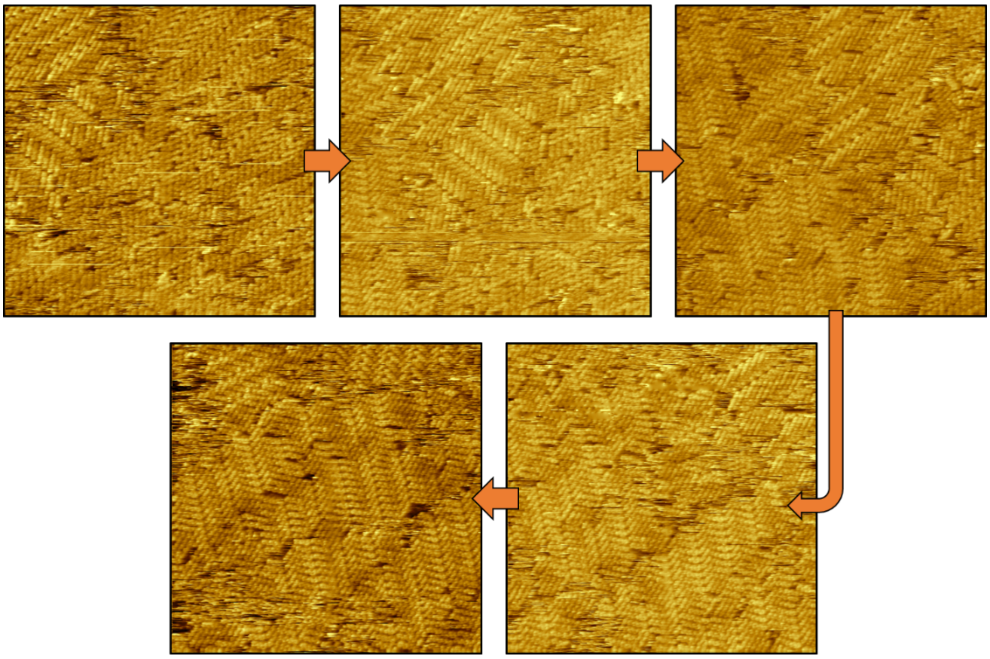

Figure S10. Real-time STM after switching to negative tip bias. The images represent subsequent scans for about 30 min imaging. Starting from the top, a large number of defects was detected within the scanned area. The molecular layer presents an Ostwald ripening with rather slow defect-healing process. Unfortunately, at these conditions the imaging frequently became highly unstable leading to no visualization of the herringbone supramolecular structure. Scanned areas: 30 nm x 30 nm; $I = -131$ pA, V = − 900 mV.

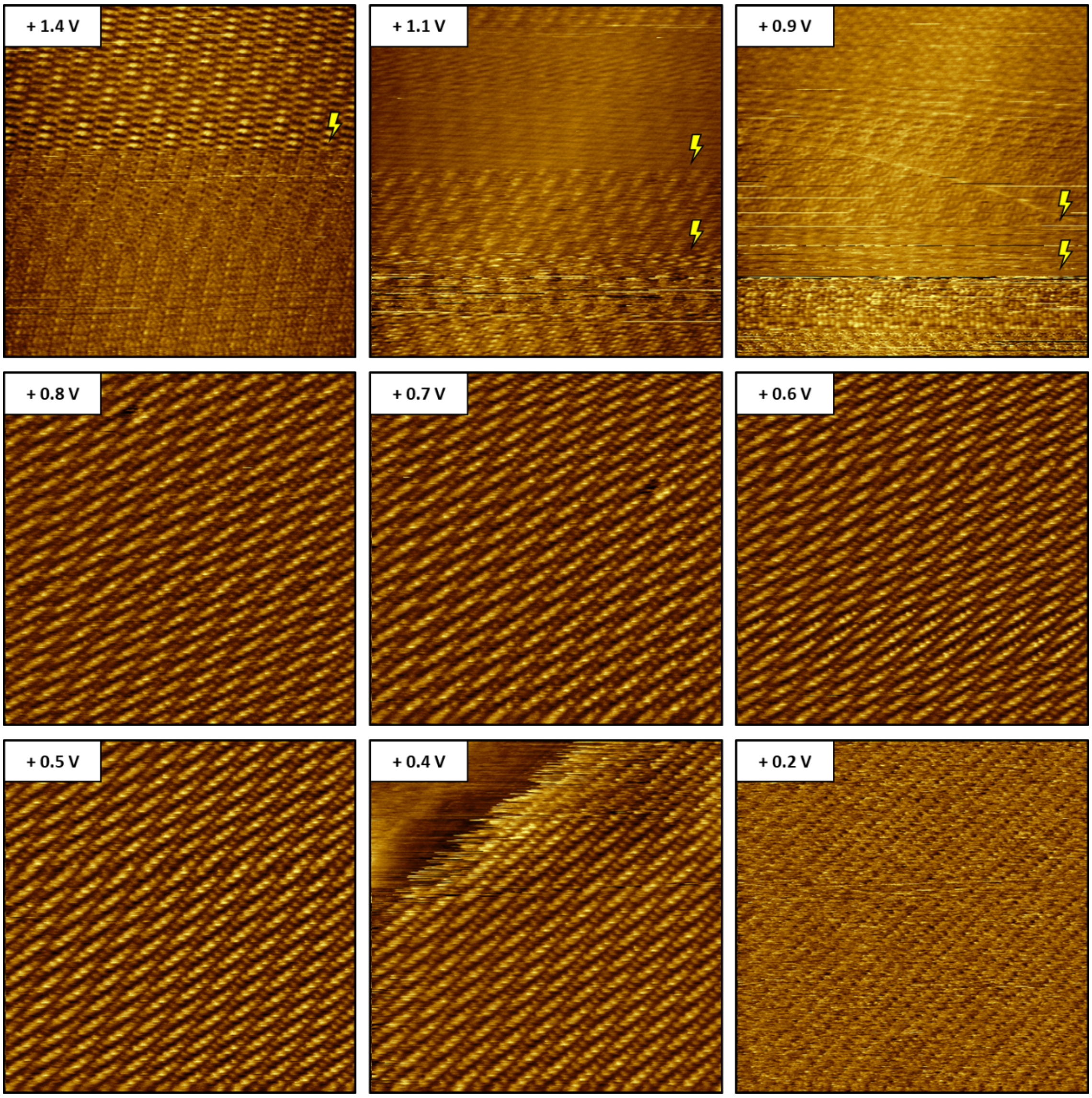

Figure S11. STM imaging performed by gradually changing positive tip bias. The insets represent the tip bias used. Starting from + 1.4 V, the same lamellar structure with Moiré pattern is visible until around + 0.2 V. Above + 1.4 V the STM became unstable and did not allow for imaging. From + 0.1 V the underlying HOPG was visible and the measurement is not reported here in the Figure. STM tip clean pulses were used in order to get better image contrast. Scanned areas: 30 nm x 30 nm.

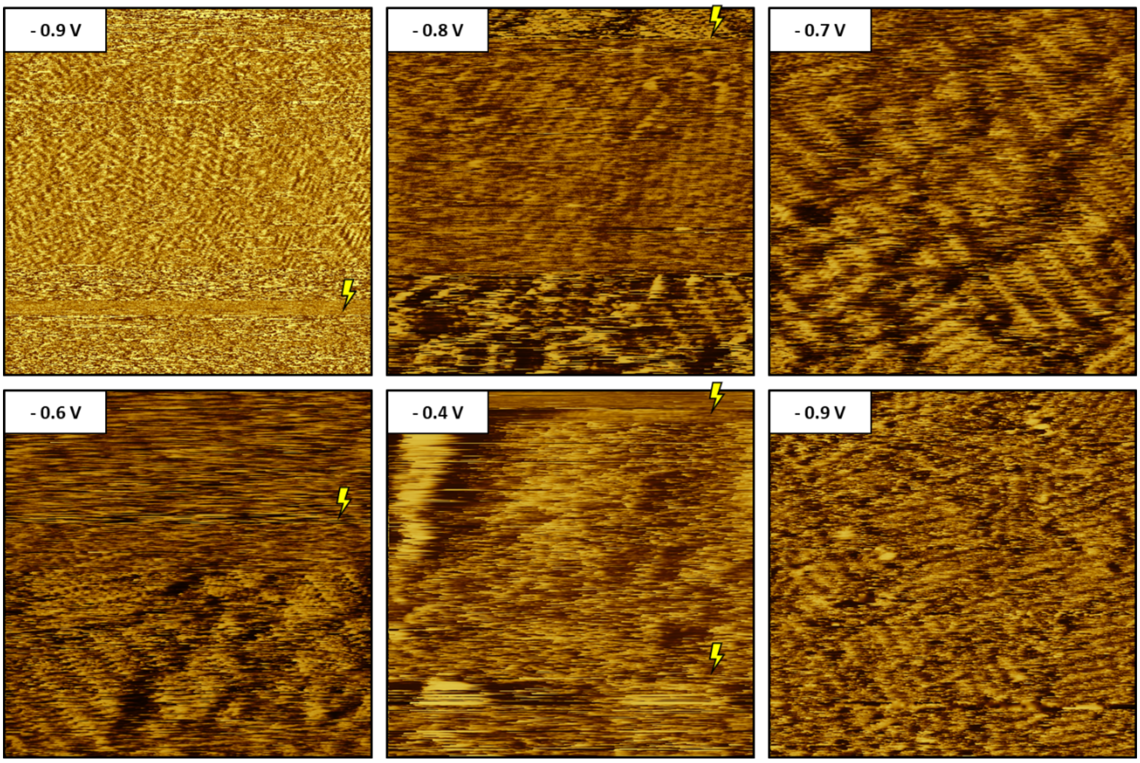

Figure S12. STM imaging performed by gradually changing negative tip bias. At – 0.9 V the new herringbone structure is clearly visible. Current image is herein used simply for a better visualization of the 2D networks. Already at – 0.8 V, the non-optimal imaging conditions resulted in tip instabilities. As for the positive tip bias, pulses were used here as well to get a better image contrast. From – 0.6 V, the supramolecular structure resulted less visible and harder to image. At – 0.4 V (or less) the supramolecular structure was completely gone. Going back to the optimal scan conditions (– 0.9 V) restored completely the 2D network. Scanned areas: 30 nm x 30 nm.

3. Molecular Modelling and Molecular Dynamics

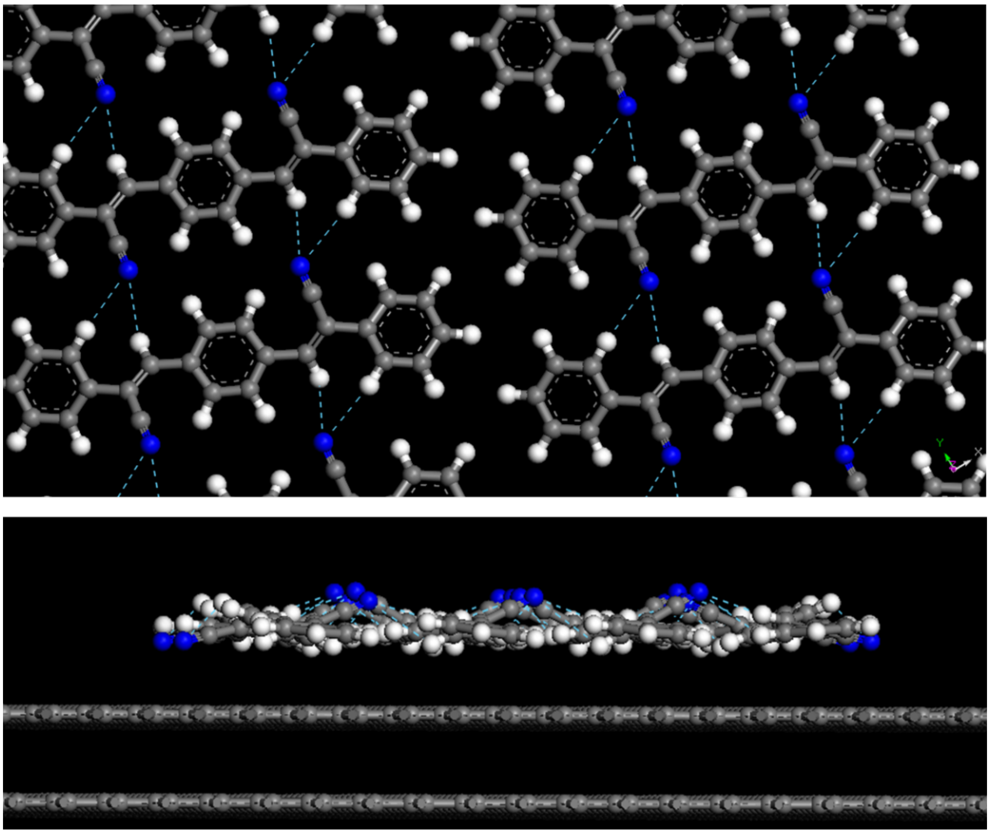

Figure S13. Molecular model of **β-CNDSB** 2D molecular network obtained by energy minimization on a graphitic surface. Hydrogen bonds are shown as blue dotted lines. At the bottom it is possible to observe the twisted conformation of the single molecules, with CN-groups pointing out of the graphite basal plane.

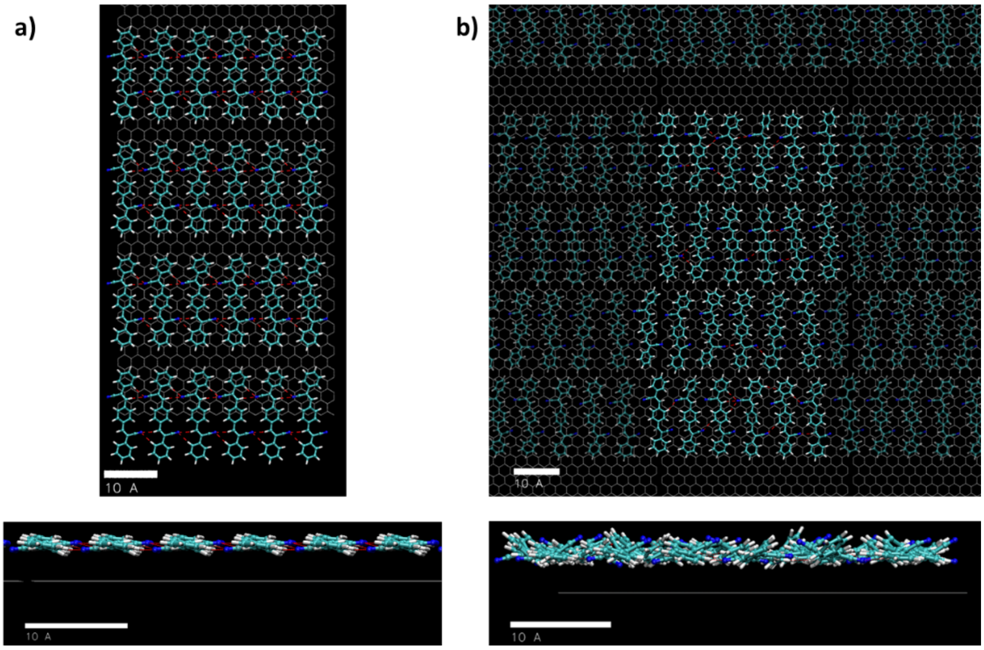

Figure S14. **a)** Minimized lamellar structure of **β-CNDSB** 2D molecular network on a graphene sheet. H-bonds are highlighted with red dotted lines. **b)** A snapshot after molecular dynamics simulations carried out at 1 ns at 300 K.

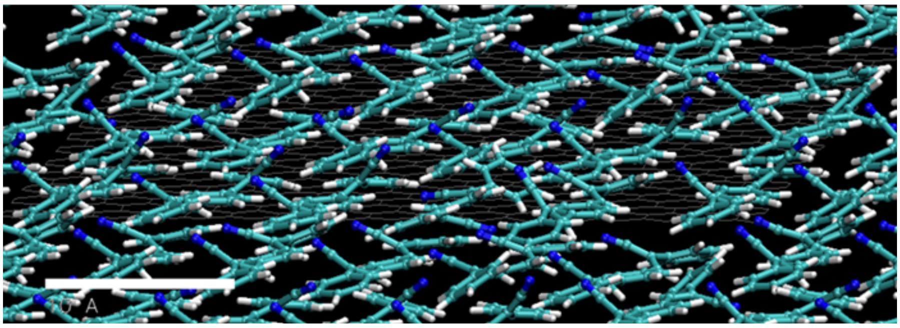

Figure S15. Molecular dynamics simulations obtained after 5 ns at 300 K of the lamellar structure carried out upon application of a negative electric field (— 2 V/nm) perpendicular to the substrate. The obtained snapshots show partial conformational isomerization of **β-CNDSB**. By applying high electric fields, CN-group are strongly tilted parallel to the electric field.

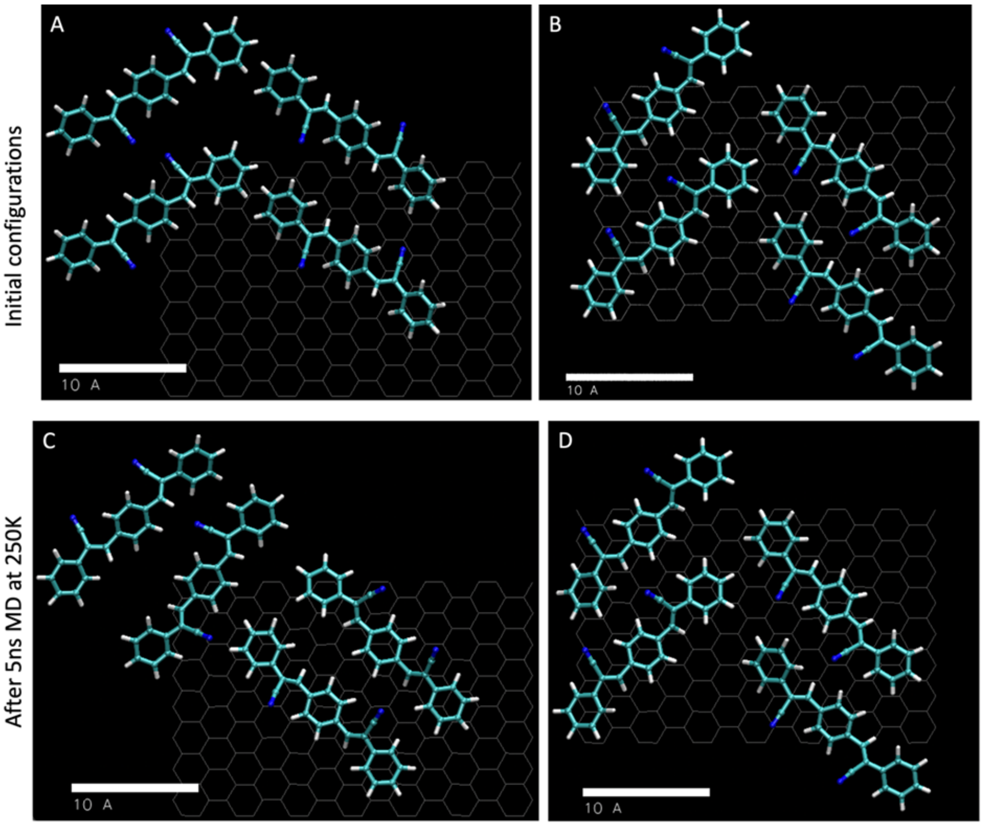

Figure S16. Comparison between the two possible conformational isomers of **β-CNDSB** disposed in the herringbone structure. After running molecular dynamics simulations for 5 ns at 250 K, only the isomer with two CN-groups pointing in the same direction resulted stable in this conformation (**B** and **D**) while the other isomer develops in a lamellar-like structure (**A** and **C**).

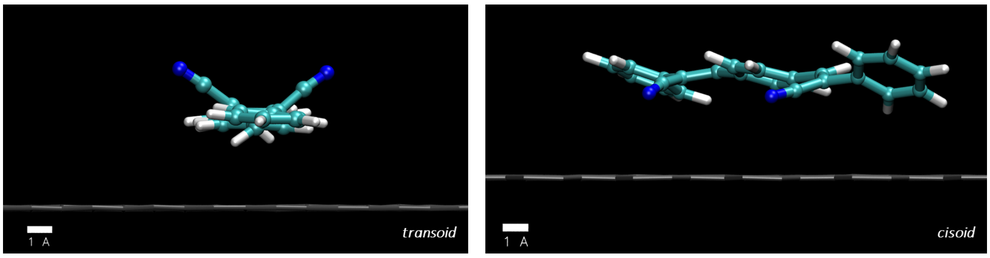

Figure S17. Lateral view of single transoid (left) and cisoid (right) molecules of **β-CNDSB** obtained by molecular dynamics calculations.

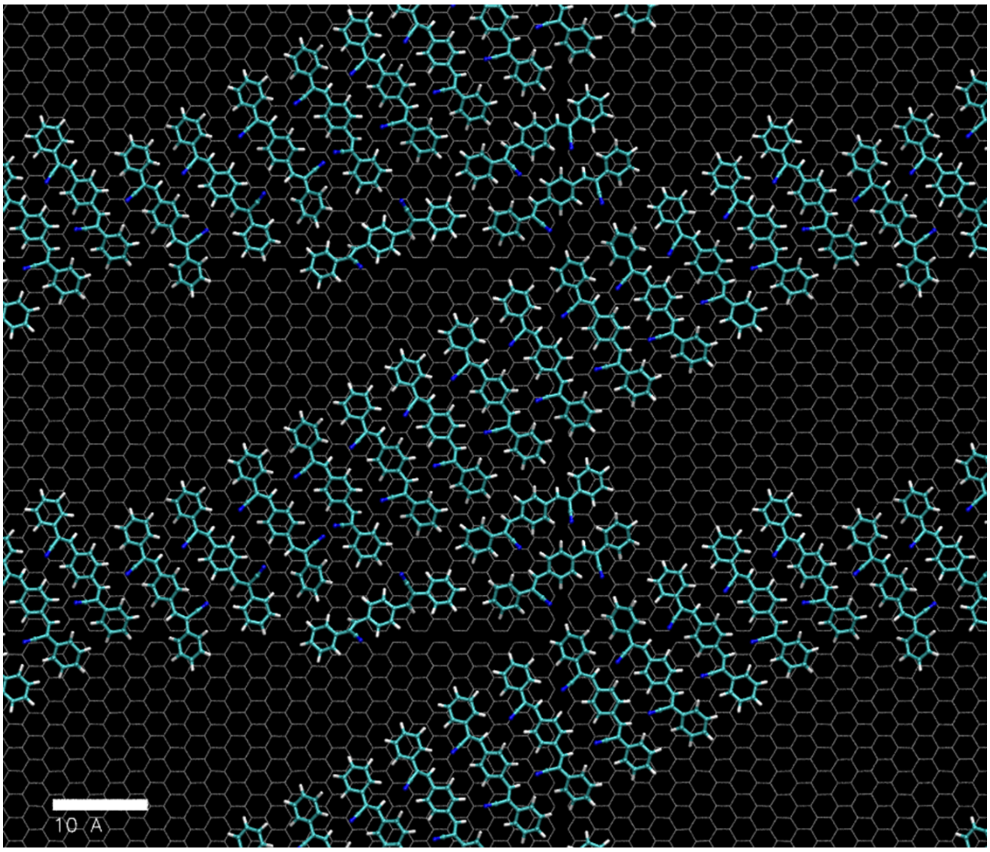

Figure S18. Partial recovery lamellar structure of **β-CNDSB** by switching the applied electric field to – 1.5 V/nm. The molecular dynamics simulation snapshot was taken after 5 ns at 250 K.

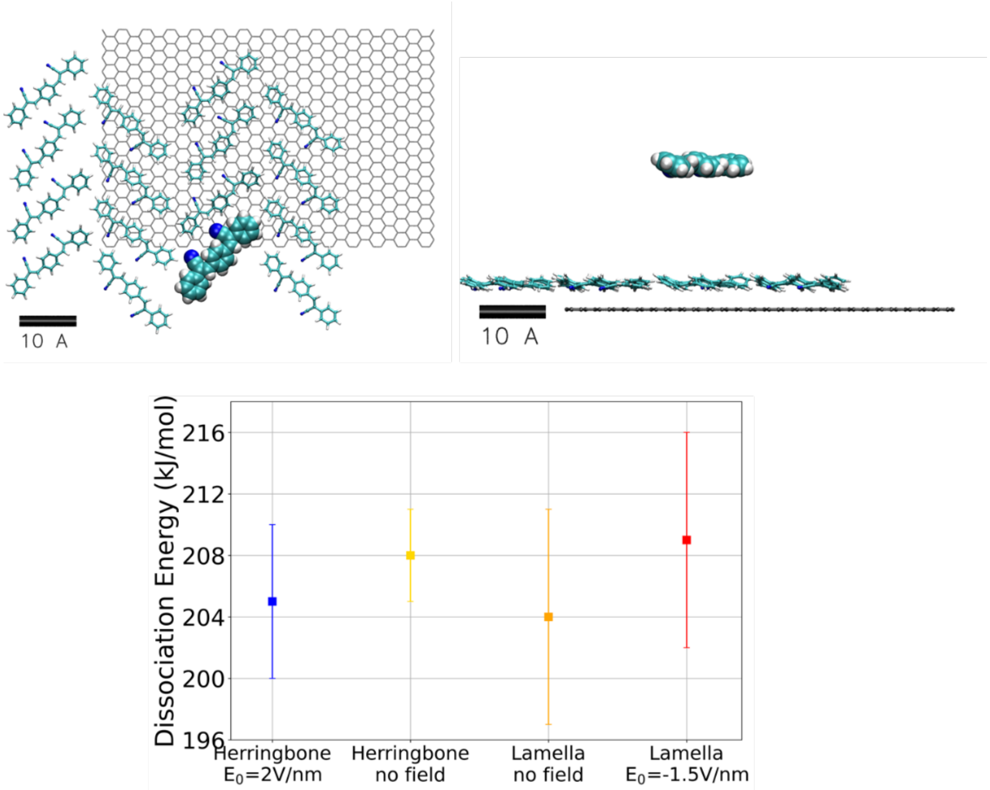

Figure S19. Above are shown the starting (left) and final (right) points during the calculations of the dissociation energies of a **β-CNDSB** in the herringbone structure. Below is reported the dissociation energy plot for all configurations and conditions. Each point represents the average over 5 dissociation plots, and the error bars as standard deviations.

4. NMR Characterization

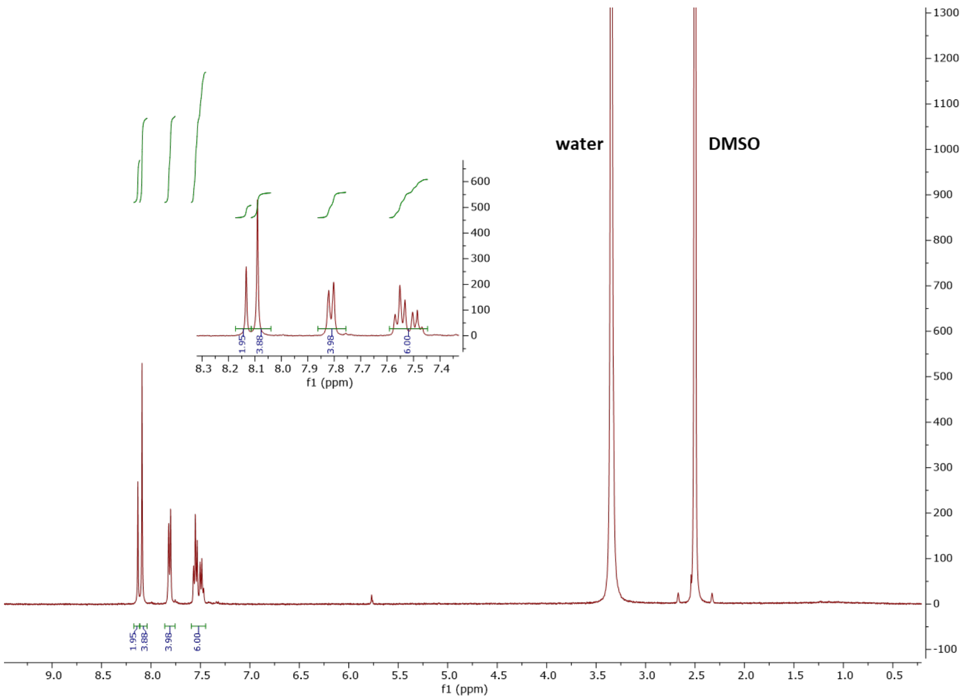

Figure S20. ^{1}H-NMR of **β-CNDSB** in DMSO-d_6

5. References

(1) Nečas, D.; Klapetek, P. Gwyddion: an open-source software for SPM data analysis. *Cent. Eur. J. Phys.* **2008**, 10 (1), 181-188;

(2) Abraham, M. J.; Murtola, T.; Schulz, R.; Páll, S.; Smith, J. C.; Hess, B.; Lindahl, E. GROMACS: High performance molecular simulations through multi-level parallelism from laptops to supercomputers. *SoftwareX* **2015**,1–2, 19–25;

(3) Vanommeslaeghe, K.; MacKerell, A. D. Automation of the CHARMM General Force Field (CGenFF) I: Bond Perception and Atom Typing. *J. Chem. Inf. Model.* **2012**, 52, 3144–3154;

(4) Vanommeslaeghe, K.; Raman, E. P.; MacKerell, A. D. Automation of the CHARMM General Force Field (CGenFF) II: Assignment of Bonded Parameters and Partial Atomic Charges. *J. Chem. Inf. Model.* **2012**, 52, 3155–3168

(5) Bussi, G.; Donadio, D.; Parrinello, M. Canonical sampling through velocity rescaling. *J. Chem. Phys.* **2007**, 126, 014101.
